# Antipsychotic Polypharmacy for Treatment Resistant Schizoaffective Disorder: A Case Report

**DOI:** 10.7759/cureus.80204

**Published:** 2025-03-07

**Authors:** Sydney E Moriarty, Paria Parhami, Jay Parikh, Linda Galicki

**Affiliations:** 1 HOLO Labs - Simulation and Educational Technology, Edward Via College of Osteopathic Medicine, Blacksburg, USA; 2 Psychiatry, Edward Via College of Osteopathic Medicine, Blacksburg, USA; 3 Psychiatry, Lewis Gale Medical Center, Salem, USA; 4 Psychiatry, Salem Veterans Affairs Health Care System, Salem, USA

**Keywords:** combination antipsychotic therapy, psychosis, schizoaffective, substance use, treatment resistant

## Abstract

This case presents a male in his early 20s with schizoaffective disorder and cannabis use disorder presenting with treatment resistance demonstrated by inadequate control of psychotic symptoms despite multiple trials of antipsychotics. The patient was experiencing positive symptoms, including hyper-religiosity, hallucinations, mood lability, agitation, and aggression. The patient and next of kin were unable to provide consent for clozapine due to concerns for monitoring requirements. The patient continued paliperidone long-acting injectable (LAI) 234 milligrams (mg) every four weeks and divalproex 750 mg twice per day (BID) and was started on haloperidol with titration to 2 mg BID. Following the addition of a second antipsychotic agent, the patient exhibited marked improvement with reduced intensity of perceptual disturbances. This case underscores the potential benefits and challenges of antipsychotic polypharmacy in addressing residual psychosis and improving functional outcomes. We also provide an overview of available evidence for and against antipsychotic polypharmacy, defined as two or more antipsychotic medications used in a patient concurrently, as well as alternative non-antipsychotic agents for treatment-resistant schizophrenia.

## Introduction

Schizoaffective disorder, a distinct nosological entity from schizophrenia, affects approximately 0.3% of the population and requires unique therapeutic considerations [1]. Additionally, schizoaffective disorder is more common in women [2]. Patients with schizoaffective disorder experience a variety of symptoms including positive, such as delusions, hallucinations, and disorganized speech, and negative symptoms such as diminished emotional expression [2]. To meet diagnostic criteria, schizoaffective disorder requires a major mood episode for majority of the illness duration, with a period of at least two weeks where mood symptoms are absent in the presence of psychotic symptoms [2,3]. Psychotic symptoms include hallucinations, delusions, disorganized thinking, or speech, and disorganized or abnormal movement. Patients may also experience negative symptoms, including difficulty in creating plans, speaking, expressing emotions, or finding pleasure [1].

In the treatment of schizophrenia, pharmacotherapy with antipsychotics plays a vital role [4]. Antipsychotics can be divided into typical and atypical antipsychotics. Atypical antipsychotics are often preferred as they have a lower risk in causing extrapyramidal side effects (EPS), such as tardive dyskinesia [5]. Furthermore, monotherapy is often preferred as it is believed polypharmacy increases the risk of side effects [4]. Notably, at least 20% of those with schizophrenia fail to adequately respond to monotherapy, posing challenges in clinical psychiatry [4]. This case presents a patient with treatment-resistant schizoaffective disorder. While there is no universally defined definition of treatment-resistant schizophrenia (TRS), some sources define TRS as patients who fail two different adequate trials of antipsychotic drug therapy [6]. Research suggests TRS is not uncommon, occurring in one-fifth to one-half of patients diagnosed with schizophrenia [6]. Clozapine is the preferred therapy for these patients as randomized trials have demonstrated clozapine’s greater efficacy compared to first- and other second-generation antipsychotics in treating TRS, though this medication requires monitoring for potential side effects including agranulocytosis [7].

## Case presentation

History and examination

This case presents a male military veteran in his 20s with schizoaffective disorder and cannabis use disorder who presented with acute exacerbation of psychosis in the context of daily cannabis use. History of substance use is important to ascertain from a patient, as it can mimic other mental disorders. The patient was discharged from the military 6.5 months earlier, after two years of service, with two previous psychiatric hospitalizations. During his prior hospitalization, he was diagnosed with schizoaffective disorder. The patient denies other medical conditions. Family history is significant for bipolar disorder in his mother. Previously, the patient had trials of lithium, divalproex, risperidone, and olanzapine without adequate control of his psychotic symptoms (Table 1).

**Table 1:** Hospitalizations course. All hospitalizations occurred during the same year. qd: daily; qhs: daily at night; BID: twice per day; PRN: as needed; q6h: every 6 hours; mg: milligram; LAI: long acting injectable

Month of Inpatient Hospitalization	September	October	November	December
Medication Changes with Medication Name and Dose	Added: Divalproex 500mg qd in morning, Divalproex 1000mg qhs, Lithium 150mg BID, Olanzapine 30mg qhs, Trazodone 50mg qhs PRN for insomnia, Hydroxyzine 50mg q6h PRN for anxiety; Adjusted: None; Discontinued: None; Continued: None	Added: Risperidone 3mg BID; Adjusted: Increased Trazodone to 150mg qhs; Discontinued: Divalproex, Lithium, Olanzapine; Continued: Hydroxyzine 50mg q6hr PRN for anxiety	Added: Loading dose Paliperidone LAI 234mg followed by 156mg, Divalproex 500mg BID; Adjusted: Decreased Trazodone 50mg qhs PRN for insomnia; Discontinued: Risperidone; Continued: Hydroxyzine 50mg q6hr PRN for anxiety	Added: Risperidone 4mg qhs as bridge to maintenance Paliperidone, Haloperidol 2mg BID; Adjusted: Increased Divalproex 750mg BID, Increased Trazodone 100mg qhs; Discontinued: Risperidone following maintenance Paliperidone; Continued: Hydroxyzine 50mg q6hr PRN for anxiety
Response/Tolerability	Readmitted the following month	Readmitted the following month	Transferred to our team	Resolution of distressing psychotic and mood symptoms

At the beginning of his hospitalization, following direct transfer from another hospital, the patient presented with visual hallucinations characterized by hyper-religiosity, including ritualistic behaviors and self-reported communication with supernatural entities. He experienced extreme mood lability with episodes of agitation and aggression, including physical aggression towards objects and verbal aggression directed towards others, that were clearly distressing to him, fluctuating with periods of calmness. His symptoms were noted to have started soon after discharge from the military and preceded Cannabis sativa use, but appeared to worsen following his initial hospitalization. 

Differential diagnosis

Patients presenting with recent intoxication of a substance, either acutely or chronically, with concomitant psychotic symptoms are challenging. When evaluating patients with psychotic symptoms, it is important to consider whether the potential underlying cause is due to an independent psychotic disorder, substance use, or a psychotic disorder exacerbated by substance use. In these clinical situations, it is important to maintain a high suspicion of substance-induced psychotic disorders. Substance-induced psychotic disorders are within the Schizophrenia Spectrum and Other Psychotic Disorders category of the Diagnostic and Statistical Manual of Mental Disorders, Fifth Edition (DSM-5) and can occur with various substances of abuse [1]. When considering schizophrenia or schizoaffective disorder in the context of substance use, effort must be made to determine the duration and circumstances of the symptoms. Substance-induced psychotic disorders can be differentiated from independent psychotic disorders if the symptoms persist longer than one month after severe intoxication or cessation and acute withdrawal, or if the symptoms are known to have preceded the usage of substances [1]. Once suspicion of an independent psychotic disorder is established, investigation must be made to delineate the psychotic symptoms’ characteristics, including frequency and history, as well as the presence of mood symptoms, such as a major depressive, manic or mixed episode. In this case, when considering the potential underlying causes of the patient's acute psychosis, we discussed cannabis intoxication. This diagnosis was not supported due to the lack of intact reality testing with concurrent visual hallucinations, as our patient was unable to differentiate internal stimuli from external stimuli and environment [1]. While our patient was using cannabis daily, upon further evaluation of history, presence of mood symptoms, and duration of the symptoms, it was determined that symptoms preceded cannabis use. In this case, diagnosis remained consistent with schizoaffective disorder given the presence of hallucinations and delusions for more than two weeks in the absence of major mood symptoms [1].

Treatment

This patient had previous trials of lithium, divalproex, risperidone, and olanzapine without adequate control of psychotic symptoms. Prior to admission, in the past, he had been treated with oral risperidone, and completed two loading doses of paliperidone long-acting injectable (LAI), a metabolite of risperidone. There is a possibility he may have been non-compliant with his medications in the past as medication level monitoring performed during hospitalizations in the past revealed subtherapeutic levels. Upon admission, divalproex dosage was increased from 500 milligrams (mg) twice per day (BID) to 1000 mg BID. The valproic acid (VPA) level was 114 mcg/ml (reference range: 50 - 100 mcg/ml). Divalproex was decreased to 750 mg BID to bring VPA level within the therapeutic range at 99 mcg/ml (reference range: 50 - 100 mcg/ml). During this time, he was also taking risperidone orally in conjunction with the paliperidone LAI and divalproex. The risperidone was increased to 4 mg daily at night (qhs) until the first maintenance dose of paliperidone was administered, then risperidone was discontinued. He received the first scheduled maintenance dose of paliperidone 234 mg based on his loading dose timing, within four weeks. Following this, the patient’s mood continued to fluctuate, and residual psychosis consistent with symptom pattern prior, persisted beyond that compatible with his stated goals. Despite the evidence suggesting clozapine would be effective given the resistant nature of this patient’s disorder, the patient and next of kin were unable to provide consent for clozapine due to concerns for frequent monitoring. This presented a unique situation of managing residual psychosis in a patient who is already taking one antipsychotic medication. The patient demonstrated tolerability and response to as-needed haloperidol for rapid symptom reduction. Upon further discussion with the patient and his next of kin, the team decided to initiate and titrate haloperidol. Haloperidol was introduced at a low dose and carefully titrated to 2 mg BID within three days to optimize therapeutic response and minimize potential side effects. The haloperidol was initiated approximately two days following the maintenance dose of paliperidone LAI. He responded well to this dosage of haloperidol and continued improving over a nine-day period. He was able to discuss his future goals, did not respond to internal stimuli, demonstrated insightfulness into his circumstances, and expressed his emotions and feelings in a calm manner. Literature has shown peak plasma concentrations of paliperidone palmitate once monthly are achieved with a median of 13 days post-injection [8]. Given the rapid improvement following haloperidol, with continued improvement following, we attributed the effects to the addition of haloperidol. The patient was discharged on paliperidone LAI 234 mg every four weeks, haloperidol 2 mg BID, and divalproex 750 mg BID with plan for outpatient follow-up. Complete metabolic panel was checked on follow-up with liver function values within normal limits. 

Outcome 

During hospitalization, the patient did not experience any side effects or extrapyramidal symptoms from his medications. Abnormal Involuntary Movement Scale (AIMS) completed prior to discharge was 0. He experienced resolution of distressing psychotic and mood symptoms with return to premorbid levels of functioning. He felt better equipped to pursue his goal of completing higher education with his G.I. bill, benefits available as the Servicemen’s Readjustment Act to assist American military veterans. While he declined specific substance use disorder treatment, he planned to abstain from cannabis, continue treatment and follow up with an outpatient psychiatrist.

## Discussion

The American Psychiatric Association (APA) guidelines provide a 1A recommendation for patients with schizophrenia to be treated with antipsychotic medications and monitored for effectiveness [9]. The APA also provides 1A recommendations for patients who have responded to antipsychotic medication, to continue to be treated with antipsychotic medications [9].

Options beyond clozapine for TRS are limited, but investigation efforts are underway. While the APA advocates for monotherapy [10], recent studies have demonstrated utility in antipsychotic polypharmacy (APP) [11]. APP is not an uncommon practice, with a frequency ranging from 19.6% to 40% [12,13]. A systematic review and meta-regression exploring global and regional trends of APP evaluating 1,418,163 participants, 82.9% diagnosed with schizophrenia, found the most common combination was a first-generation antipsychotic and second-generation antipsychotic at 42.4% [13]. Additionally, research has shown a decreased risk for psychiatric hospitalization in patients on low-dose and high-dose APP [11]. Further research is needed exploring other potential combinations in addition to the safety and efficacy. A review of agents as potential adjuncts to antipsychotic use in treatment-resistant schizophrenia is provided below, and each agent’s pertinence to the treatment of the patient in the presented case.

Research has demonstrated that lamotrigine is superior to placebo in clozapine augmentation for patients with treatment resistance [14]. Although, when considering the need for rapid symptom reduction, the length of time taken for titration to maintenance dose may be around eight weeks as titration speed is limited by potential for developing a life-threatening rash [15]. While Jang et al. reported the efficacy of a rapid titration protocol reaching 200 mg within 11 days, there were limitations to this study including a small cohort and study design [15]. While these results were promising, the small cohort evaluated coupled with the known consequences of up-titrating lamotrigine too quickly with our patient's need for rapid symptom reduction was not enough for us to consider transitioning our patient off divalproex, another anticonvulsant, to trial lamotrigine.

Minocycline has shown promise as a potential adjunctive medication for schizophrenia [16], proposed for its anti-inflammatory, anti-apoptotic, and antioxidant properties [17]. A meta-analysis, including 10 trials with a total of 415 patients, demonstrated greater efficacy in treatment with minocycline adjunctively versus placebo [17]. However, some research has demonstrated minocycline was effective in reducing total symptoms, negative symptoms, and general symptoms, while not showing an improvement in positive symptoms or cognitive domains [17]. Our patient was primarily experiencing positive, psychotic symptoms and for these reasons, we decided to continue looking for an alternative.

Topiramate is another medication frequently used in antipsychotic augmentation and suggested in literature for schizophrenia. In a meta-analysis of 12 randomized controlled trials, Okuyama and colleagues reported that topiramate-augmentation therapy showed superiority in decreasing overall symptoms when compared to antipsychotic alone or placebo plus antipsychotic [18]. Furthermore, topiramate augmentation was superior in reducing positive and negative symptoms [18]. However, this research had limitations, including a small number of patients included in the trials, a short follow-up period and lack of investigation of optimal dosage [18]. For these reasons, and the patient’s current therapy of divalproex, another anti-convulsant, we decided to forgo a topiramate trial.

Amisulpride, a benzamide antipsychotic with D2 and D3 dopamine receptor selectivity, is another augmenting agent being explored [19]. An open-label trial of 40 patients with schizophrenia or schizoaffective disorder found that, of the 29 subjects who completed the trial, 34.5% had greater than 50% response to the add-on therapy of amisulpride [19]. Other research studies have explored combinations of amisulpride add-on to an antipsychotic demonstrating statistical significance [20,21]. While these results were encouraging, our goal was to continue the LAI medication and these studies either excluded patients on LAIs or studied a different medication combination.

The patient's clinical trajectory highlights the effective use of haloperidol in acute symptom stabilization, emphasizing the importance of individualized treatment plans for TRS. With the addition of a typical antipsychotic agent, the patient demonstrated marked improvement with reduced intensity of perceptual disturbances and subsequent reduction in distress caused by these experiences. He also showed improvement of negative symptoms including positive, future-oriented thinking and an increase in mood stability.

## Conclusions

Our case demonstrates rapid and adequate treatment of psychosis through the utility of a typical antipsychotic agent in the setting of concurrent atypical antipsychotic LAI and anticonvulsant in a patient with treatment resistance. While polypharmacy may utilize a combination of an atypical and typical antipsychotic agent, broader application requires careful consideration due to the potential side effects and limited evidence currently available. Limitations of this study include lack of long-term data with follow-up, evaluating for side effect development and continued efficacy of the medication regimen, as well as other possibly confounding variables including other environmental factors. Other limitations include lack of standardized symptom scales, quantifying the medication response prior and following administration, as well as other alternative treatment options to clozapine that were not included in this article.
